# Functional Nucleic Acid-Based Live-Cell Fluorescence Imaging

**DOI:** 10.3389/fchem.2020.598013

**Published:** 2020-12-11

**Authors:** Yutong Zhang, Yulin Du, Yuting Zhuo, Liping Qiu

**Affiliations:** ^1^Molecular Science and Biomedicine Laboratory (MBL), State Key Laboratory of Chemo/Bio-Sensing and Chemometrics, College of Chemistry and Chemical Engineering, College of Biology, Aptamer Engineering Center of Hunan Province, Hunan University, Changsha, China; ^2^NHC Key Laboratory of Birth Defect for Research and Prevention, Hunan Provincial Maternal and Child Health Care Hospital, Changsha, China

**Keywords:** live-cell imaging, aptamer, DNAzyme, fluorescence probe, functional nucleic acid

## Abstract

Cell is the structural and functional unit of organism. It serves as a key research object in various biological processes, such as growth, ontogeny, metabolism and stress. Studying the spatiotemporal distribution and functional activity of specific biological molecules in living cells is crucial for exploring the mechanism governing life. It also facilitates the elucidation of pathogenesis, clinical prevention and disease theranostics. In recent years, the fluorescence imaging technique has been greatly exploited for live-cell imaging. However, the development of molecular probes has lagged far behind. Functional nucleic acids (FNAs), for example, aptamer and DNAzyme, possess special chemical and/or biological functions, hence severing as promising molecular tools for cellular imaging. The current mini review focuses on the applications of FNAs in live-cell fluorescence imaging.

## Introduction

Cell, as the fundamental unit of life, was composed of a highly complex but ordered structure. It could be elaborately subdivided into functionally distinct compartments and contained various biological molecules, e.g., small molecules, nucleic acids and proteins. The capability to monitor specific molecules with high spatiotemporal resolution would be significant to unravel the mechanism underlying related physiological processes (Cooper and Hausman, 2000).

Live-cell imaging was emerging as a significant strategy to study the cellular physiological process, enabling capture of dynamic information for specific molecules (Ebrahimi et al., 2020). As one of the most attracted imaging techniques, fluorescence imaging has been widely applied in areas of biomedical and biological research (Sokolov et al., 2003; Imamura et al., 2018). Specially, advances in fluorescence microscopy had allowed great progresses in the study of cellular biology. On the other hand, the application of such imaging techniques might be confounded by the lack of appropriate molecular probes.

In recent decades, various small molecules (Liu et al., 2018), peptides (Tung, 2004) and nucleic acids have been developed as fluorescence probes and utilized in the realm of live-cell analysis. Particularly, nucleic acid-based probes, were booming as attractive candidates owing to their advantages of good biocompatibility, low immunogenicity, easy synthesis, and convenient modification.

Typically, it has been discovered that nucleic acids can bind with specific molecules or function as catalysis like protease (Lu and Liu, 2006). These nucleic acid molecules with specific functions were regarded as functional nucleic acids (FNAs), in which aptamer and DNAzyme were considered as two typical representatives. Several decades in the past have witnessed the exponential rise in the application of FNAs in biological and biomedical research.

## Functional Nucleic Acids (FNAs)

In 1990, Andrew D. Ellington et al. postulated the concept of aptamer, a term derived from the combination of “aptus” in Latin and “meros” in Greek (Ellington and Szostak, 1990; Tuerk and Gold, 1990). Aptamers were single-stranded oligonucleotides (DNA or RNA) that bound to target cargos through folding into specific secondary/tertiary conformation. The process in which aptamers bound to the target cargos was similar to the interaction between antibody and antigen, thus aptamers were referred as “chemical antibody” (Fang and Tan, 2010). Compared with antibodies, aptamers had several advantages including easy modification, reversible manipulation, low immunogenicity, fast penetration and so on.

As reported by Gerald. F. Joyce et al. in 1994, DNAzymes were a member of metalloenzyme family and functioned as catalysis in the presence of co-factors (Breaker and Joyce, 1994). They could modulate DNA and RNA cleavage, DNA ligation, peroxidase activity and so on. Their catalytic ability could be activated in conjunction with specific co-factors, such as metal ions. Studies over the past several decades have provided various applications of DNAzymes in bioanalysis and biomedicine (Scott, 2017).

Both aptamers and DNAzymes were screened by the *in vitro* competitive process termed Systematic Evolution of Ligands by Exponential Enrichment (SELEX). In the process of SELEX, target molecules were incubated with the nucleic acid library. Then unbounded nucleic acids were washed away, and bounded ones were extracted and amplified through polymerase chain reaction (PCR). At last, nucleic acids that had a high affinity with target molecules were isolated and enriched through repeated cycles. During the past decades, a wide range of SELEX techniques have been developed. Especially, Cell-SELEX was developed by using intact living cells as the selection target, with no need to involve prior knowledge of the membrane protein (Shangguan et al., 2006), providing a novel technology for screening molecular tools for cell analysis.

The past few decades have witnessed the applications of FNAs-based fluorescent probes in live-cell imaging. In this mini review, we focused on the application of FNAs for fluorescence imaging at the cellular and subcellular level.

## Live-Cell Fluorescence Imaging With Functional Nucleic Acid-Based Probes

### Cell Recognition and Detection

Cell recognition with high specificity was a primary requirement in precision medicine, which mainly relied on recognition of proteins expressed on the cell membrane. Membrane proteins accounted for one third of all human proteins, and represented nearly 50% of therapeutic targets (Overington et al., 2006). One major advantage of Cell-SELEX was to maintain the native state of membrane proteins during the selection process.

By conjugating these aptamers with fluorescent dyes, target cells could be directly visualized with fluorescence imaging (Cerchia and de Franciscis, 2010). For example, Wu et al. identified an aptamer XQ-2d for specifically targeting a pancreatic ductal adenocarcinoma cell line, PL45, with an affinity of 82.5 nM (Wu et al., 2015). By linking a fluorescein (FAM) dye to aptamer QX-2d, the specific fluorescence imaging of PL45 was realized. Additionally, owing to the plasmon resonance, gold nanoparticles aggregated with accompany of a spectrum shift, resulting in a color change from red to blue/purple. Based on this principle, Medley et al. developed aptamer-functionalized AuNPs to enable cancer cell detection with naked eyes (Medley et al., 2008). Despite positive preliminary results, these aptamer probes were “always on,” potentially leading to a high background signal. To improve the imaging sensitivity, Shi et al. designed an activable aptamer probe (AA2) based on the conformational changes (Shi et al., 2011). In the absence of the target, AA2 was maintained in a harpin conformation where the fluorophore was approaching to the quencher, resulting in fluorescence quenching. Upon binding with the target, acute lymphoblastic leukemia cell line, CCRF-CEM, the hairpin conformation of AA2 was unfolded, resulting in a fluorescence restoration. As such, recognition-induced signal from “off” to “on” could be achieved, thus enhancing the signal-to-noise ratio of target cell imaging both *in vitro* and *in vivo*.

Normally, cells were highly heterogeneous, it was difficult to achieve precise cellular identification through a single-parameter detection. For example, human epidermal growth factor receptor-2 (HER2) protein was not merely expressed on cancer cells, but also expressed on normal cells with a relatively low content. Herein, it was challenging to distinguish cancer cells from normal cells in terms of HER2 only. To improve the accuracy of cellular identification, multiparameter-based cell recognition strategies were developed. In 2014, Wang et al. used three different aptamers which were separately labeled with three different fluorophores to develop a multicolor imaging method for distinguishing different types of cancer cells (Wang et al., 2014). Taking advantages of three detection parameters on individual cells, the accuracy of cellular identification could be remarkably improved. In addition to targeting cells, exosome, a cell-excreted vesicle that maintained similar membrane characteristics could serve as an alternative marker for fluid biopsy of cancers. Jiang et al. designed an aptamer-functionalized AuNP platform capable of identifying exosomes with naked eyes (Jiang et al., 2017). On sensing the target exosome, aptamer would fold into specific tertiary conformation and thus dissociate from AuNPs, leading to particle aggregation accompanied with a change of absorption spectrum. Furthermore, by using multiple aptamers, the profiling of exosome could be realized. To achieve simple operation and low-cost testing, Liu et al. reported an aptamer-based thermophoretic sensor for collecting and detecting exosomes from minute amounts of serum (Liu et al., 2019). Finally, in the combination with machine learning algorithm, the membrane protein information of exosomes was utilized for early profiling detection of six types of cancer.

**Figure 1:**
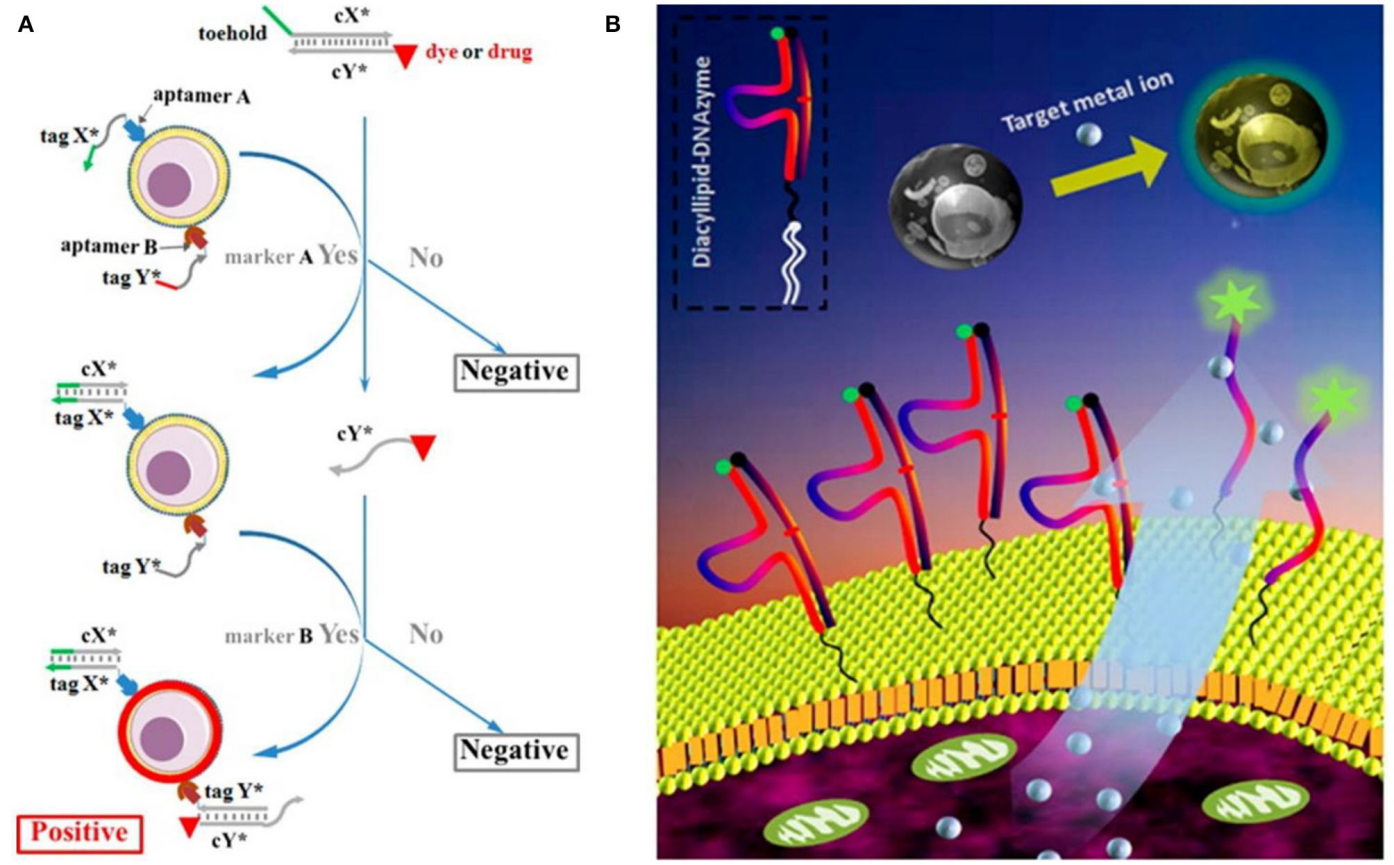
**(A)** Scheme of DNA-based logic platform for multiparameter cancer cell recognition (You et al., 2015); **(B)** Scheme of diacyllipid-conjuageted DNAzyme fluorescence probe (Qiu et al., 2014). Reproduced with the permission of American Chemical Society.

To achieve intelligent diagnosis, multiple aptamers were used to construct DNA circuits for analyzing the expression pattern of cancer membrane proteins. For example, You et al. (2015) designed a programmable DNA-based nanoplatform and constructed both “AND” and “OR” logic gates to improve the recognition specificity of CCRF-CEM (Figure 1A). In subsequent studies, Chang et al. introduced the hybridization chain reaction (HCR) to amplify the recognition signal generated in the logic gate operation (Chang et al., 2019).

Notably, one major advantage of aptamers was their reversible recognition capability, which made them flexible tools for cell type-specific capture and release. Kacherovsky et al. screened an anti-CD8 aptamer via Cell-SELEX and used this aptamer to specifically capture CD8^+^ T cells from peripheral blood (Kacherovsky et al., 2019). The captured T cells were released through the strand displacement reaction with addition of a complementary DNA, offering a traceless way for CD8^+^ T cell isolation with high purity and high yield. They also demonstrated that CD8^+^ T cells isolated with this strategy had the same functional effector as that isolated with the antibody-based strategy. Furthermore, Li et al. thoroughly studied different kinetic mechanisms governing the disassociation of aptamers from the cellular surface using complement DNA (cDNA), toehold-labeled cDNA (tcDNA) and single-stranded binding protein (SSB), laying a solid foundation for aptamer-based capture and isolation of target cells (Li L. et al., 2019).

### Subcellular Imaging

#### Cell Membrane-Based Microenvironment Imaging

The cellular microenvironment referred to a local and dynamic surrounding around the cell that exerted a vital influence on the cellular behavior through transmission of either physical, chemical, or biological signals. It played a critical role in regulation of various cellular functions, such as metabolism, reproduction, differentiation, apoptosis, and communication (Joyce and Pollard, 2009). Real-time monitoring of specific molecules in such a microenvironment would provide essential information to understand the mechanism governing many biological processes.

In 2011, Zhao et al. covalently modified an aptamer probe on the surface of mesenchymal stem cells for real-time monitoring of platelet-derived growth factor (PDGF) in the cellular microenvironment (Zhao et al., 2011). In despite of considerable interest, the covalent modifications of the cell membrane could potentially affect the natural cellular state, which would limit their applications, to some extent. To address this issue, non-covalent cellular modification methods, especially the hydrophobic interaction-based strategy, have emerged as attracted alternatives. In this light, a variety of amphiphilic DNA probes, which were composed of a hydrophobic fragment, a DNA fragment and a linker in between, were developed for cell surface engineering.

For example, Qiu et al. designed and synthesized a diacyllipid-conjugated DNAzyme probe for real-time monitoring of target metal ions in the cellular microenvironment (Qiu et al., 2014). Based on the hydrophobic interaction between the diacyllipid fragment and the phospholipid bilayer, this amphiphilic probe could be effectively anchored onto the cell membrane without affecting the cellular activity (Figure 1B). In addition to excellent membrane decoration efficiency, it was endowed with several advantages, including good biocompatibility, simple operation, and general applicability. The results also demonstrated that this membrane-anchored DNAzyme probe was able to dynamically monitor the cellular secretion process of target ions. In order to enhance the membrane-anchoring stability of DNA probes, Li et al. continued to develop an amphiphilic three-dimensional (3D) DNA probe using DNA tetrahedrons with three hydrophobic vertexes as the scaffold. This 3D DNA probe showed nearly 100-fold improvement on the membrane-anchoring stability, compared with that of the linear one (Li J. et al., 2019).

In addition, membrane-anchored DNA probes have been used to study the dynamic nature of the plasma membrane. You et al. used amphiphilic DNA probes as the building block and developed DNA-based reaction circuits to investigate the membrane locomotion through toehold-mediated strand displacement. The transient encounters between the membrane-anchored DNA probes could translate the membrane movement into fluorescent signals, thus providing a powerful platform to study molecular interactions of the cellular interface (You et al., 2017).

To improve the spatial resolution of imaging, super-resolution imaging techniques have been actively applied for fluorescence imaging of receptor proteins on the cell membrane. In 2006, Hochstrasser et al. developed the point accumulation for imaging in nanoscale topography (PAINT), based on the instant collision between the probe and the target, and achieved significant improvement on the spatial resolution of fluorescence imaging (Sharonov and Hochstrasser, 2006). Since the working principle of PAINT mainly relied on the molecular thermal motion, the imaging resolution was rarely linked with the density of the probe. DNA, owing to its predictable and controllable hybridization, showed great potential in PAINT. In 2010, Jungmann et at. developed a technique of DNA-PAINT (Jungmann et al., 2010), where a DNA docking strand was bounded to the target molecule, and a fluorescent dye-labeled DNA imaging strand contained a complementary sequence of the docking strand. When the imaging strand was in instant contact with the docking strand, a scintillating fluorescence signal would be generated. Aptamer, because of its small size, quantitative labeling as well as fast diffusion, held great potential in this aspect. In 2018, Strauss et al. (2018) utilized aptamer to realize the super-resolution imaging for seven different proteins for both cellular membrane and cytoplasm.

#### Cytoplasm Imaging

The cytoplasm was a primary site for cell metabolism and many biochemical reactions. Studying the dynamic distribution of specific components in the cytoplasm would definitely offer important information for study of various cellular processes. FNAs have attracted wide attention in intracellular fluorescence imaging with their excellent specificity, easy modification, and high programmability. Conversely, nucleic acids, which were negatively charged, couldn't easily penetrate the cell membrane due to the electrostatic repulsion, thus limiting their applications in the cytoplasm. Recently, great progress has been made to overcome this drawback via development of many cellular delivery methods.

##### Inorganic nanomaterial-based delivery

Inorganic nanomaterials with large surface area and rigid structures have been widely used in nucleic acid delivery. As a notable example, in 1996, Mirkin et al. reported spherical nucleic acid (SNAs) composed of inorganic core and DNA shell (Mirkin et al., 1996). Rosi et al. then proved that the cellular uptake efficiency of SNAs could reach as high as 99% (Rosi et al., 2006). Furthermore, Seferos et al. reported live-cell imaging of mRNA based on SNAs functionalized with recognition DNA and reporter DNA (Seferos et al., 2007).

Apart from 3D inorganic nanoparticles, 2D nanosheets, have been used as a delivery carrier for nucleic acids through π-π interactions. Wang et al. reported a Graphene Oxide (GO)-based strategy for the cellular delivery of fluorophore-labeled aptamer that could specifically recognize ATP (Wang et al., 2010). On sensing intracellular ATP, the aptamer could form a specific tertiary structure and then liberate from the GO. In this way, the fluorescence would be recovered for indicating the presence of ATP. By combining with unique physical and chemical features of inorganic nanomaterials, not only could the cellular uptake efficiency of FNAs be improved, but also multi-functions could be realized.

##### DNA nanostructure-based delivery

In recent years, an interesting phenomenon was frequently reported that DNA molecules can effectively enter living cells through forming rigid 3D nanostructures. In addition, formation of DNA nanostructures could protect DNA molecules from nuclease digestion, which would be beneficial for the reduction of false-positive signals. In 2017, He et al. designed and synthesized DNA tetrahedron nanotweezers (DTNTs) based on Fluorescence Resonance Energy Transfer (FRET) for live-cell imaging of tumor-related mRNA (He et al., 2017). After the cellular entry, DTNTs could bind with target mRNA to “turn off” the FRET signal. With introduction of the entropy-driven amplification strategy, the target-responsive fluorescence signal could be effectively amplified to improve the imaging sensitivity (He et al., 2018).

##### Internalized aptamer-based delivery

**Figure 2:**
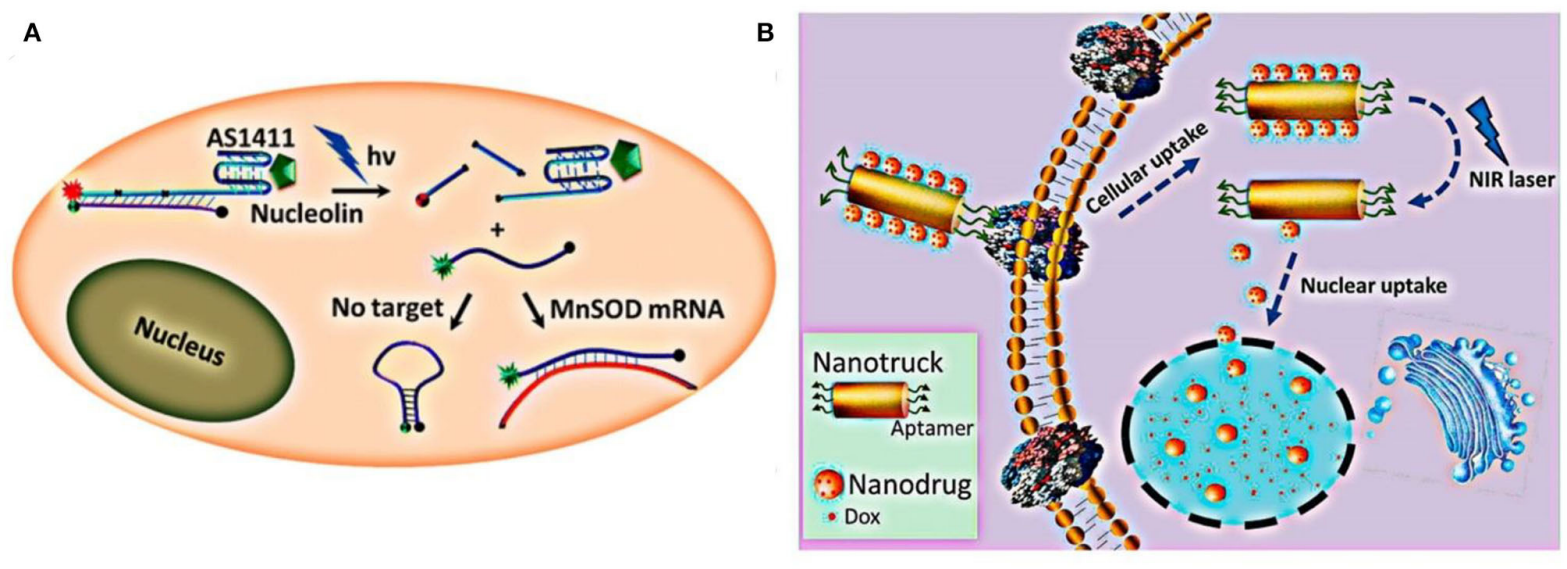
Strategies for intracellular imaging of biological molecules **(A)** AS1411-based internalized strategy for mRNA imaging (Qiu et al., 2013); **(B)** Smart nanodrug delivery system with nuclear uptake (Qiu et al., 2015); Reproduced with the permission of American Chemical Society.

Aptamers screened from Cell-SELEX could specifically bind with the membrane proteins, and some of them could be driven into the cells by the translocation behavior of the target protein, thus providing an excellent ligand for cell-specific delivery (Yoon and Rossi, 2018). For example, aptamer AS1411 could specifically bind with nucleolin that were overexpressed on the membrane of many cancer cell lines. Based on the shuttle behavior of nucleolin between the cytoplasm and the nucleus, AS1411 could be efficiently internalized by cancer cells. By using AS1411 as the targeting ligand, Qiu et al. developed a self-delivered and photo-controlled molecular beacon (MB) for intracellular mRNA analysis (Qiu et al., 2013). After entering the cell, the PC linker was cleaved upon light irradiation, thus releasing MBs to sense the target mRNA accomplished with fluorescent recovery (Figure 2A).

#### Nucleus Imaging

Nucleus was the control center of the cell. The live-cell imaging of specific components in the nucleus would be of great importance. Based on the nucleolus-targeting property of AS1411, Sun et al. designed an aptamer-functionalized Ag cluster for nucleus imaging (Sun et al., 2011). In addition, aptamer Ch4-1 screened by Shen et al. was validated to bind the nucleus with high affinity (K_d_ = 6.65 ± 3.40 nM), which was used for identification of dead cells through nucleus imaging in an apoptosis assay (Shen et al., 2019). To make the cellular delivery smarter, Qiu et al. developed an aptamer-functionalized photo-responsive nano-assembly system (Qiu et al., 2015). After the nano-assembly was internalized into the cell, small-sized nanodrugs could be accumulated in the nucleus upon exposure of near-infrared (NIR) light (Figure 2B). Their results demonstrated that this delivery system held great promise in overcoming the P-glycoprotein-mediated multidrug resistance (MDR).

## Summary and Outlook

The past 20 years have witnessed the rapid advances of FNAs in interdisciplinary areas of chemistry, biology and medicine. Based on the *in vitro* screening technology, a large number of FNAs have been screened against diverse specific target molecules, providing excellent tools for biological and biomedical applications. Meanwhile, taking advantage of high programmability, good biocompatibility, easy synthesis and convenient modification, FNAs have gained considerable attention and shown great potential in live-cell study. On the other hand, FNAs were still in the preliminary stage, challenges remained for practical applications. First, FNAs could be easily digested by nucleases, their biostability could be a primary concern. Many strategies, such as utility of artificial bases and terminal-blocked scheme, have been developed to solve this issue though, there was still plenty of room for improvement. Second, FNAs that possessed high specificity and high affinity to their targets were relatively lacking. The innovation of the SELEX technique to generate high-performance FNAs would be desirable. Third, the recognition mechanism of most aptamers with their target molecules remained unclear, which would apparently hinder the application scopes. This was a major reason why only several aptamers, e.g., anti-ATP and anti-thrombin aptamers, have been frequently used in fundamental research. Meanwhile, to achieve subcellular imaging with high spatiotemporal resolution, FNAs capable of targeting different organelles should be exploited.

## Author Contributions

YZha drafted the manuscript. LQ guided and amended the manuscript. YD and YZhu helped to review the manuscript. All authors contributed to the manuscript.

## Conflict of Interest

The authors declare that the research was conducted in the absence of any commercial or financial relationships that could be construed as a potential conflict of interest.
